# Spontaneous rupture of the common hepatic duct associated with acute pancreatitis: a case report

**DOI:** 10.1186/s13256-017-1283-6

**Published:** 2017-06-21

**Authors:** Makram Moussa, Wissem Triki, Omar Karray, Ines Marzouk, Bouchoucha Sami

**Affiliations:** 1 Department of General Surgery, Habib Bougatfa Hospital, Bizerte, Tunisia; 2 Medical School of Tunis, Tunis El Manar University, Tunis, Tunisia; 3 Department of Diagnostic and Interventional Imaging, Mongi Slim University Hospital Marsa, La Marsa, Tunisia

**Keywords:** Common bile duct, Spontaneous rupture, Peritonitis, Pancreatitis, Computed tomography, Cholangiography

## Abstract

**Background:**

Rupture of the common bile duct is a life-threatening condition, usually observed after a trauma or in association with choledocholithiasis or an obstructive tumor of the bile duct. However, a spontaneous rupture of the common bile duct is a rare entity.

**Case presentation:**

We report a new observation of a spontaneous rupture of the common bile duct, associated with biliary peritonitis and pancreatitis, in a 15-year-old North African girl. Etiological aspects, specificities of clinical presentation, means of diagnosis, as well as surgical and perioperative management are discussed.

**Conclusions:**

The diagnosis of spontaneous rupture of the common bile duct is a challenge for both radiologist and surgeon. Beyond the difficulty of diagnosis, which requires radiological exploration, management of the subsequent biliary peritonitis involves urgent surgery, life-supporting measures, and close monitoring.

## Background

Spontaneous rupture of the common bile duct (CBD) is a rare cause of biliary peritonitis. The severity of such a condition is related to the systemic complication of the peritonitis, the difficulties of perioperative management, and the high rate of mortality due to a delay in accurate diagnosis and appropriate life-support measures.

## Case presentation

A 15-year-old North African girl with no past medical history presented with fever and diffuse abdominal pain with vomiting for 3 days. A clinical examination found an altered general condition with 38 °C fever and peripheral signs of shock. Abdominal palpation revealed a diffuse guarding; there was no jaundice or other clinical signs of cholestasis.

Blood tests showed a total leukocyte count of 25,000 cells/mm^3^ with a predominance of neutrophils. Her hemoglobin level was 16 g/dl, C-reactive protein was 300 mg/ml, and creatinine was 1.69 mg/dl. There was cholestasis with high levels of conjugated bilirubin. Her amylase and lipase were respectively 423 U/l (normal 20 to 80) and 1152 U/l (normal 0 to 190).

**Fig. 1:**
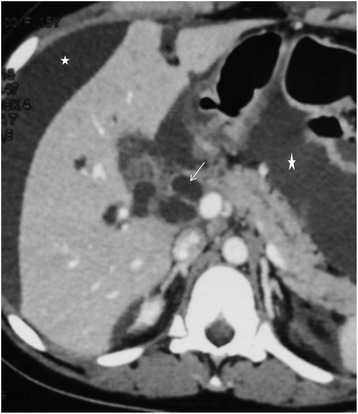
Axial computed tomography showing a dilation of the main bile duct (*arrow*) and a peritoneal fluid effusion (*asterisk*)

An abdominal ultrasound (US) followed by an abdominal computed tomography (CT) scan showed a dilation of the biliary tree; her choledochus was 17 mm with no evidence of obstacle or gallstones. A free intraperitoneal fluid was also associated (Fig. 1). Her pancreas was increased in size. She was diagnosed as having biliary peritonitis, even though imaging did not reveal lithiasis or hydatid cyst. She was admitted to the surgical intensive care unit. She was treated with antibiotic therapy (cefotaxime 1 g three times per day, gentamicin 160 mg daily) and metronidazole 500 mg three times per day. A fluid replacement was done until the onset of diuresis.

**Fig. 2:**
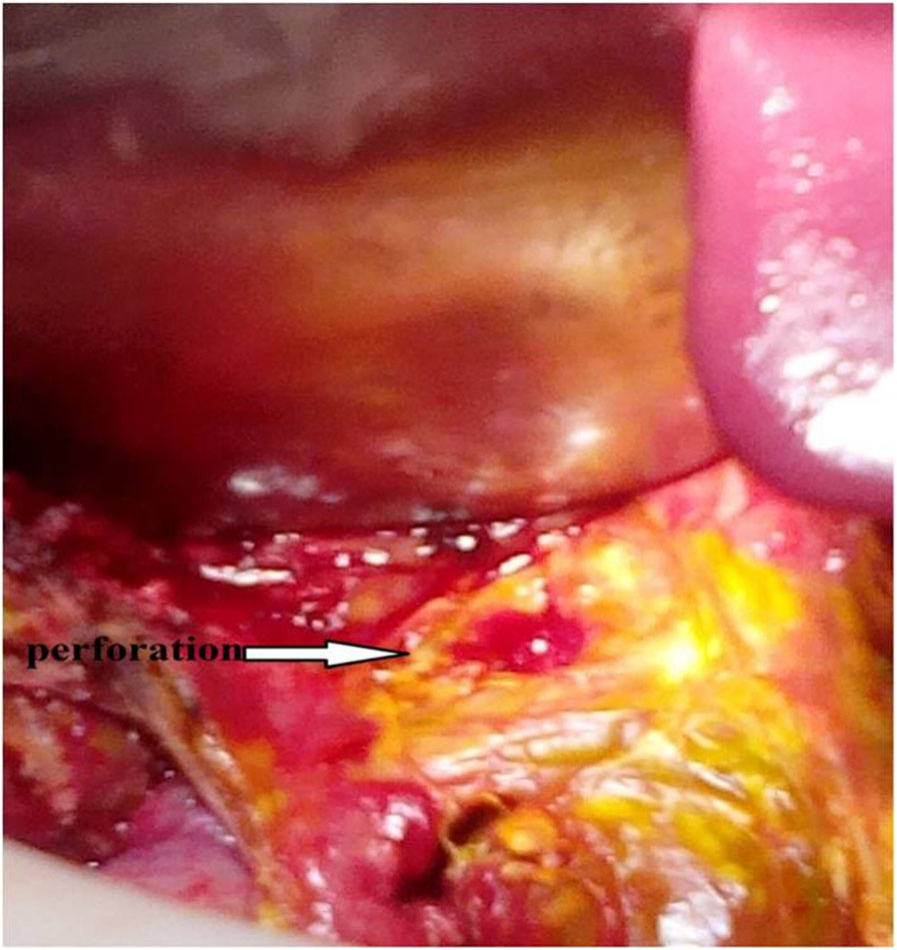
Intraoperative view showing the common hepatic duct perforation (*arrow*)

She underwent an emergency surgery with a midline laparotomy. There was a generalized biliary peritonitis related to a 1.5 cm perforation of the anterior face of her CBD (Fig. 2). Cholangioscopy exploration of her choledochus did not find any lithiasis. Her pancreas was swollen and inflammatory. A plentiful peritoneal lavage, followed by a cholecystectomy and reparation of the biliary perforation on a T-tube drain, was performed.

**Fig. 3:**
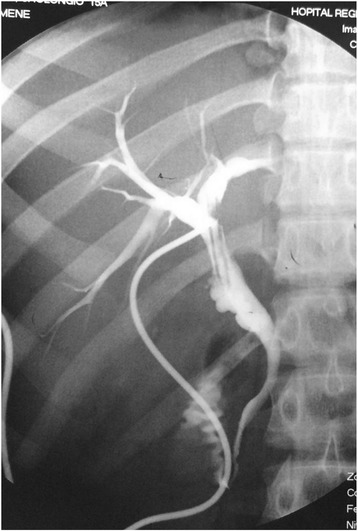
Postoperative cholangiography

**Fig. 4:**
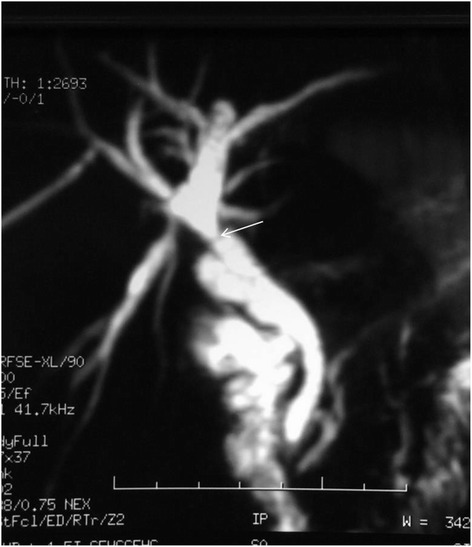
Magnetic resonance cholangiopancreatography performed 6 months after surgery showing a moderate biliary dilation among a stricture (*arrow*)

After surgery, the T-tube average flow was 300 ml per day. On the 14th postoperative day, a cholangiography showed a normal aspect of the biliary tree and massive duodenal opacification (Fig. 3). She was discharged on the 28th postoperative day. The T-tube was removed on the 42nd postoperative day. Postoperative magnetic resonance cholangiopancreatography (MRCP; Fig. 4) showed an extrahepatic biliary stricture with moderated dilation of her intrahepatic bile ducts. There was no biological cholestasis. Regular follow up in the out-patient clinic did not reveal any clinical, biological, or radiological modifications.

## Discussion

Spontaneous perforation of the biliary tract (SPBT) is a serious and rare entity, which is most often seen in childhood. Since its first description by Freeland in 1882 [1–4], only around 150 cases have been reported worldwide [5].

The average age of occurrence is 4 years, with frequency peak at 6 months [3]. Pathogenesis of the perforation is still unclear. It may be related to various mechanisms [4, 6], such as the ductal high pressure upstream from an obstacle, parietal necrosis due to thrombosis of the intramural vessels, and parietal infection favored by ductal stasis. Parietal necrosis may also be related to the activation of a pancreatic reflux. In our case, we did not measure the amylase in the bile. Most of the previously reported cases are related to the presence of a lithiasis, representing 75% of the causes.

For our patient, the biliary perforation is probably multifactorial. It may have been caused by a pseudocystic dilation of her CBD, which represents 1.8 to 7% of the etiologies [7, 8]. A spasm of the Oddi sphincter may have caused a reflux of the pancreatic secretions. It seems to be the most involved factor in our patient, as a pancreatic reaction was observed on admission and no calculi were found on intraoperative CBD exploration. A postoperative sphincterotomy was not performed because the Oddi sphincter spasm was transient as a magnetic resonance cholangiopancreatography showed a regression of bile ducts dilation.

SPBT is more frequent in the junction between the cystic duct and the CBD, which is a zone of congenital weakness [9]. In childhood, clinical presentation differs, depending on the age of the patient. Newborns mainly have abdominal distension, jaundice, and underdevelopment; whereas babies and children generally present with peritonitis and a severe sepsis [5]. In our observation, the patient initially complained of a painful abdominal distension; the perforation is located in her CBD, which is rarely reported in the literature.

In such situations, urgent surgery is indicated, with a rigorous peritoneal lavage, associated with repair of the CBD preferentially using a T-tube drain. If the biliary perforation is not identified, drainage of the abdominal cavity will lead to a spontaneous healing of the biliary tract. Complications may be observed while spontaneous healing takes place, mainly stenosis of the CBD.

Mortality reaches 67% when patients do not undergo surgery quickly, perforation is not recognized, or in patients presenting other comorbidities [10]. Although symptoms evolved for 3 days for our patient, urgent surgery and perioperative life-supporting measures allowed a favorable evolution.

## Conclusions

SPBT in children is a rare condition. Diagnosis is evoked on the association of clinical arguments and radiological aspects, and is usually confirmed in the perioperative exploration. Urgent surgical treatment is the most influential prognostic factor in multidisciplinary management.

## Abbreviations

CBD: Common bile duct
CT: Computed tomography
MRCP: Magnetic resonance cholangiopancreatography
SPBT: Spontaneous perforation of the biliary tract
US: Ultrasound
